# Young People’s Perspective on Discussing Intersectionality and Diversity During Psychological Therapy: A Qualitative Analysis in a Specialist Child and Adolescent Mental Health Service

**DOI:** 10.1177/13591045261419862

**Published:** 2026-01-20

**Authors:** Hanan K. S. Khalaf, Nada Abou Seif, Zoë Maiden

**Affiliations:** 1 Institute of Psychiatry, Psychology and Neuroscience, 34426 King’s College London, London, UK; 2 National (Child & Specialist services), South London & Maudsley NHS Trust, London, UK

**Keywords:** child mental health, diveristy, psychological therapy, qualitative methods, identify

## Abstract

**Background:**

Research suggests that National Health Services have continued to report health inequalities in access to treatment, care and outcomes. Individuals with diverse identities continue to report feeling like their specific needs are not addressed. The current study explored young people’s perspective of discussing diversity (faith/religion, gender, sexual orientation, disability) as part of therapy within a National and Specialist CAMH service.

**Methods:**

Semi-structured interviews were conducted to explore young people’s perspectives of discussing diversity characteristics as part of therapy. 10 young people were interviewed, and a thematic analysis was employed.

**Results:**

Results revealed 4 main themes including (1) perspectives on diversity and identity (2) therapeutic relationship, (3) integrating diversity in therapy and (4) Barriers to exploring diversity in therapy.

**Conclusion:**

Findings of the present study demonstrates the complexities of approaching diversity in therapy and provide insights into how clinicians can adapt therapy when supporting young people with these concerns.

## Plain Language Summary

People from diverse backgrounds often feel that their specific needs are not addressed in mental health services. This study looked at the experiences of young people discussing aspects of their identity including religion, gender, sexuality and disability, during psychological therapy. 10 young people were interviewed to gather their perspectives. The study found 4 main themes (1) how identity is shaped by different aspects of diversity (2) the importance of the relationship between the therapist and the young person (3) how diversity can be discussed in therapy (4) reasons why young people may be reluctant to discuss diversity in therapy. Results of the study showed the complexities of discussing diversity in therapy and showed ways in which mental health professionals can better support young people to discuss diversity during therapy.

## Introduction

### Diversity in Mental Health Treatment

Disparities in healthcare are complex, with some proposing that conventional psychotherapy may be irrelevant or harmful towards individuals with diverse characteristics (e.g., culturally diverse, Hall, 2001). This is because conventional psychotherapeutic interventions do not address systemic barriers or discrimination often faced by individuals from diverse backgrounds (Hall, 2001). It is important for clinicians to be equipped with skills in discussing diversity as part of therapy as studies highlight clinicians encountering clients with diverse characteristics throughout their careers.

### Note on Terminology

Terminology around diversity is continually evolving, reflecting cultural and societal shifts (Castania, 2003). Diversity in a broad sense is about recognition and valuing differences, and the term “*diversity*” has been prevalently adopted in a wide range of mental health/research settings (Bradby & Brand, 2015; Park et al., 2024). In this study, the term *diversity* is used to capture the wide range of differences among individuals and groups (e.g., gender identity, sexual orientation) and is not limited to individuals who have been historically marginalised but all individuals, recognising the multifaceted ways in which identities and experiences intersect. The use of this terminology aims to highlight complexities in identities while reflecting current evidence and discussions in the literature.

### Diversity Characteristics and Mental Health

Different diversity characteristics have varying impact and significance across society. For example, the longstanding history of discrimination through pathologising lesbian, gay and bisexual identities need to be acknowledged when considering modern day understandings of mental health in sexually minoritised groups (Bartoli & Gillem, 2008). Similarly, research in gender diverse individuals (e.g., trans) commonly identifies barriers to care including discrimination or lacking professionals’ competence (Canvin et al., 2022; Pitts et al., 2009). Overall, there is a clear need to address these issues in mental health services to ensure that these groups feel able to access and engage in support.

Religion is understood by many to be a source of coping and healing for both therapists and clients (Koenig, 2012), yet incorporating discussions around religion as part of therapy remains challenging for professionals (Astrow et al., 2001). In a study by Elkonin et al. (2014), clients emphasised issues around lack of training in addressing religion including feeling uneasy about therapists crossing boundaries when integrating religion into therapy. However, it is important to address religion as part of therapy as such topics may contribute to a client’s distress (Johnson et al., 2007). Thus, religious individuals may be less likely to seek mental health support or may feel misunderstood in a therapeutic setting.

Disability also needs to be considered in therapy (Simpson & Thomas, 2015). Disability is multifaceted, with ongoing debate about whether conditions like Autism Spectrum Disorder (ASD) should be considered as a disability (Lester et al., 2014). Nevertheless, research suggests significant disparities in accessing therapy for disabled individuals even though these individuals are more likely to report mental health concerns compared to non-disabled individuals (Choi et al., 2020). These issues leave disabled individuals without appropriate resources to manage their wellbeing, resulting in marginalisation within mental health care systems.

Despite recognition of these differences, research suggests that National Health Services (NHS) have continued to report health inequalities in access to treatment, care and outcomes (Lowther-Payne et al., 2023; Patel & Hanif, 2022). Individuals with diverse identities (e.g., ethnicity, gender, sexuality, etc) report feeling like their specific needs are not addressed (Owen & Khalil, 2007) and continue to struggle accessing support due to these concerns. Yet, these individuals continue to be overrepresented in mental health settings due to a combination of structural inequalities and complex difficulties from a cumulation of unmet needs. Thus, it is critical to increase awareness, knowledge and understanding of these diversity issues in clinical work to ensure inclusive and effective care for individuals with varied backgrounds.

### Cultural Humility in Addressing Diversity

Having cultural humility and understanding issues related to diversity is seen as a key aspect in providing effective mental health care. Past research demonstrates that most practitioners will have worked with at least one client with an identified diversity characteristic (e.g., sexual minority; Lyons et al., 2010). Neglecting these issues can lead to inappropriate care and bias potentially causing harm to clients (Moleiro et al., 2018). Yet, many practitioners admit to having little training in working with diverse clients despite being open to diversity issues (Moleiro et al., 2018).

It is also important to consider the impact that addressing these diversity factors may have on the therapeutic relationship. The therapeutic relationship has strong supporting evidence and is regarded as an essential factor for positive therapy outcomes (Carter, 2007; Lambert & Archer, 2007). However, unique issues relevant to the therapeutic relationship may arise for people who identify with diverse characteristics such as misunderstandings (Vasquez, 2007). This can be problematic as therapists with low sensitivity to these issues may respond inappropriately, potentially fostering mistrust and compromising the therapeutic alliance.

### The Present Evaluation

Previous work done by Sarr (2024) looked at experiences of ethnic minoritised young people (YP) in a National and Specialist CAMH service. This study highlighted the unique perspectives and challenges of YP and the impact of these issues on their identity and mental health. However, there remains a gap in research investigating how other diversity factors are understood and discussed with YP.

To extend on this work, the current study explored YP’s perspective of discussing diversity factors (faith/religion, gender, sexual orientation, disability) as part of therapy within a National and Specialist CAMH service. This paper acknowledges the multifaceted nature of diversity. Frameworks such as Burnham’s Social GGRRAAACCEEESSS model (Burnham, 2018) highlights the breadth of characteristics that may impact individuals lived experiences and interactions with mental health services. However, intersectionality (Crenshaw, 2019) emphasises the dynamic and interconnected ways in which identities are mutually shaping individuals’ experiences. The present study prioritises gender identity, sexual orientation, religion and disability as focal domains due to their relevance to clinical practice and evidence of disparities in mental health access and outcomes. These domains were selected not to exclude other aspects of diversity but to enable an intersectional exploration into how multiple identity domains shape therapeutic experiences. The research aims were as follows:

• What are experiences of YP during psychological therapy, with consideration of their relevant diversity characteristics (faith, gender orientation, sexual orientation, disability)?
• What factors could benefit YP with relevant diversity characteristics during psychological therapy?

Key study factors were operationally defined as:

1. **Religion/faith:** Religion is belief in a god or gods and the activities that are connected with this belief, such as praying or worshipping in a building such as a church or temple.
2. **Gender identity:** Gender identity is each person’s internal and individual experience of gender. It is a person’s sense of being a woman, a man, both, neither, or anywhere along the gender spectrum.
3. **Sexual orientation:** Sexual orientation is the emotional, romantic, or sexual attraction that a person feels toward another person.
4. **Disability:** A disability is any condition of the body or mind (impairment) that makes it more difficult for the person with the condition to do certain activities and interact with the world around them

## Methods

### Setting

This study was conducted within a National & Specialist Child and Adolescent Trauma, Anxiety and Depression (TAD) clinic in the South London and Maudsley NHS Trust. The TAD clinic operates as a tier 4 specialist service, supporting children and adolescents with complex mental health concerns. The service offers a range of care including diagnostic assessment, training, consultation and psychological therapy, primarily specialist Cognitive Behavioural Therapy (CBT).

### Design

A qualitative design was employed to identify study aims and prioritise participants’ subjective experiences. Participants took part in semi-structured interviews to obtain their perspectives of discussions around diversity factors and relevant considerations during therapy.

### Participants

Purposive sampling was employed, with the research team actively seeking out participants. Inclusion criteria include (1) discharged from the TAD clinic in the last 18 months (2) consent given to be contacted for research purposes.

Participants were recruited by identifying YP with the assistance of TAD clinicians and through screening of client records. Several individuals (*n* = 15) were deemed ineligible due to various reasons (e.g., not having given consent to be contacted for research, not currently engaging in therapy or presenting with clinical risk). Eligible individuals were contacted and sent the information sheet with some not replying (*n* = 8) and others declining to participate (*n* = 4). 10 individuals met the inclusion criteria and consented to participate. Demographic information can be seen in Table 1.

**Table 1.** Demographic Information, *n* = 10

Characteristics		Frequency (%)
Disability	Yes^a^	4 (40%)
	No	6 (60%)
Religion	Religious affiliation	4 (40%)
	No religious affiliation	6 (60%)
Gender identity	Cisgender male	1 (10%)
	Cisgender female	8 (80%)
	Trans female	1 (10%)
Sexual orientation	Heterosexual	2 (20%)
	Sexual minority	8 (80%)
Age (range)	15–19	
Ethnicity	White-British	6 (60%)
	Black/Black British-Caribbean	3 (30%)
	Black/Black British-Ethiopian	1 (10%)

^a^Of the 4 who identified as having a disability, 3 participants noted their Autism Spectrum Disorder (ASD) as a form of disability. Within these 4 participants, 3 had a formal diagnosis of ASD. Out of the 10 participants interviewed, 8 had a formal diagnosis of ASD.

Participants were provided with a detailed information sheet outlining the purpose, procedures and potential risks/benefits of participation. Written informed consent was obtained from all participants prior to the interviews. For participants under the age of 16, parental consent was obtained to comply with ethical requirements.

### Semi Structured Interviews

Previous literature (Sarr, 2024) was used to guide the development of interview questions to ensure that responses aligned with research aims.

The full interview guide can be seen in Supplemental materials. This was revised following discussions with service users (*n* = 3) who agreed to screen interview questions.

### Procedures

Interviews were conducted by the first author (H. K.) through a mixture of online Microsoft team meetings (*n* = 7) and face to face interviews (*n* = 3). This was guided by participants’ preferences. Prior to the start of the interviews, participants were asked questions on their knowledge of the study’s key terms to establish a shared understanding regarding the operational definitions of these terms. The interview protocol was also adapted based on participants’ experiences. For segments of the interview where participants answered “*no*” to having discussed specific diversity factors as part of their therapy, these were omitted from the interview process to maintain relevance and avoid discomfort.

### Data Analysis

Data were analysed using codebook thematic analysis (Braun & Clarke, 2023). Through familiarisation of the transcripts and research aims, an initial codebook was developed. Coding was conducted through NVivo (Lumivero, 2023) and utilised an inductive and semantic approach. The codebook was iteratively refined and potential themes were conceptualised. Potential themes were reviewed against the original dataset to ensure meaningful patterns were captured across participants’ accounts. Themes and subthemes were then defined and revised to highlight the data they represented.

In keeping with best practice of qualitative research, researcher’s positionality was acknowledged. Only the first author (H. K.) conducted the analysis and had no direct clinical work with the study’s sample. Their professional background provided relevant context in interpreting participants’ experiences. To minimise bias, the researcher ensured the coding framework was maintained and revised following consultation with co-authors of the paper who are experienced in qualitative research. Additionally, the researcher ensured reflective note taking throughout the analysis to document decision making.

## Results

**Figure 1.**
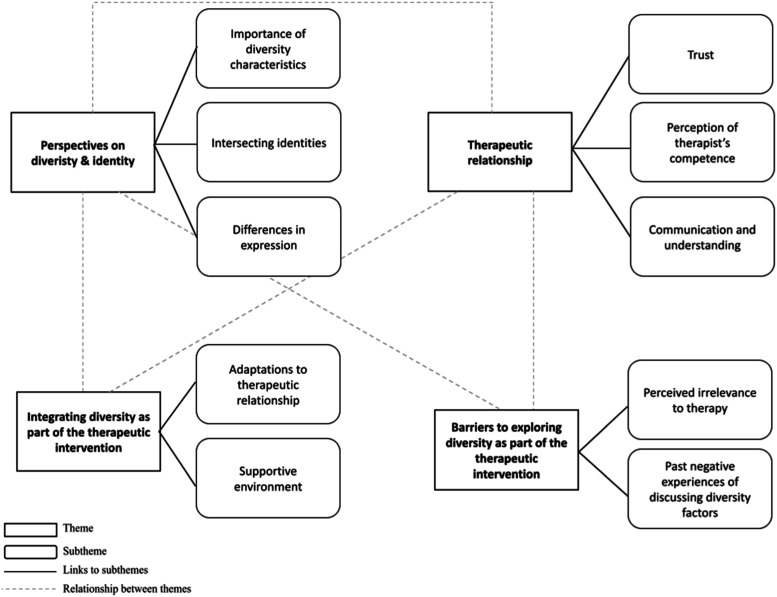
Main themes and subthemes

The analysis revealed 4 main themes (1) Perspectives on diversity and identity, (2) Therapeutic relationship (3) Integrating diversity in therapy and (4) Barriers to exploring diversity in therapy, which are explored in the following section. Figure 1 depicts the main themes and subthemes identified and the relationships between them.

### Perspectives on Diversity & Identity

This theme summarises how YP place importance on relevant diversity characteristics and its impact on their experiences of therapy. Most participants (70%) regarded gender identity as an important aspect of their self-identity while fewer participants (50%) related to the significance of religion as part of their identity.

Because well…I think it [religion] can be like a way to guide people like to be a better person, it’s like a moral compass **(P6, White British, Religious affiliation)**

Well it’s the way people see me, it’s who I am as a girl and yeah I think that’s affects a lot of different parts of my life **(P2, Black British- Caribbean, Cisgender female)**

Although some participants did not find the study terms personally significant, they recognised the importance of these diversity factors. Similarly, one participant recognised the importance of diversity factors in engaging in relationships and understanding others, despite not seeing certain diversity characteristics as defining themselves.

I don’t see it [gender identity] as a defining characteristic about myself. But again, I think it’s a kind of important thing to know. It makes relationships and other’s understanding you easier... **(P1, White British, Cisgender male)**

Participants spoke about how certain diversity factors can affect the way they relate to others and the impact of this on how they practise religion. For example, one participant spoke about constraints of traditional gender norms as part of their religion. Another participant spoke similarly on the intersection of gender identity and cultural expectations in that these factors cannot be neatly separated.

Its just like I was saying about the gender stuff. It’s just who I am and actually they all relate really. I as a girl can be straight bisexual or something else and well that changes how you relate to others or even how I practise my religion… like I know I would struggle to be the [religious identity] if I were bisexual or gay. **(P6, White British, Heterosexual, Cisgender female)**

..they [key study terms] should be blended together because, like… like, you know, gender identity in your culture, like to an extent sometimes goes hand in hand. because it’s like you have to think about how this will seem in your culture or even just norms of your race or ethnicity. **(P2, Black/Black British- Caribbean, Cisgender female)**

Participants also emphasised the differences in expression of these factors and how expressions of each characteristic can vary within the individual. Specifically, one participant emphasised avoiding assumptions based on stereotypes and reflected on differences in expressions within families.

Everyone practises it differently, I guess. And like for example, the way I practise my religion is completely different to like the rest of my family than and it’s just like being aware of those differences and just being careful to not make any assumptions. **(P3, White British, Religious Affiliation)**

### Therapeutic Relationship

The therapeutic relationship was seen as an important component for engaging in meaningful discussion around diversity factors. One participant drew from previous negative experience to highlight that lacking trust in the therapeutic relationship limited their ability to be open with their therapist. Thus, the therapeutic relationship can have an influence on participants’ willingness to address diversity factors as part of therapy.

Like if you’re expecting it to be a safe space, you need to trust the person but in some spaces that I have experienced in the past, I wasn’t able to do that, not like with my therapist here. In the past, I spoke with other therapist and they would made light of my situation which it isn't like… it’s very demeaning… **(P7, Black/Black British- Ethiopian, Cisgender female)**

Participants highlighted the importance of therapists not making assumptions of their identity and giving space to explore this based on their preferences. This allowed for participants to feel respected and enabled them to bring discussions into the therapeutic space when appropriate and based on their preferences.

I really appreciate how like she didn’t expect me to have a label or a title on anything. And because things like that are very fluid and it’s, you know, just right here and there, and it wasn’t like it was like my, like, my therapy was focused solely on that and solely focused on diagnosing me with this or that, I would have felt really uncomfortable with that. Sometimes you just wanna talk things out and you don’t wanna diagnosis for it… **(P2, Black/ Black British- Carribbean, Sexual minority)**

It was a…it wasn’t like very in your face about it. She [TAD therapist] wasn’t like “because you’re religious, we have to change everything”. It was a very understanding approach, and I didn’t feel pressured to like, talk about my religion or to act. And like, I guess the stereotypical way on how Christians are viewed, I was never expected to act in this way. I was able to still be myself and still be authentic. **(P4, White British, Religious affiliation)**

A key element of this theme relates to participants’ sense of their therapist competence with dealing with issues related to the study terms. Therapists’ skills and experiences with navigating diversity was integral at fostering trust for participants. This perceived competence allowed for participants to engage with topics relevant to diversity with more confidence and delve into discussions more meaningfully.

I mean I felt comfortable bringing it up because I knew [therapist] could like help me with it. We didn’t really talk about it until I felt comfortable with her and she had told me she’s dealt with like my issues so I knew she was like experienced and could like…you know...like handle it. **(P2, Black/Black British- Caribbean, Sexual minority)**

### Integrating Diversity in Therapy

This theme relates to how therapists modified therapy to reflect and respect the unique needs of participants. One participant indicated how discussions around the study terms, specifically sexual orientation, were integrated into trauma therapy. This suggests that the therapist was able to acknowledge and address how this diversity characteristics may influence the way in which the individual processes their experiences.

Yes. again, I think it might have been early on in our sessions, but we also talked about it [sexual orientation] in the context of the trauma and how it was like set in terms of goal setting in terms of whether I wanted to be more comfortable with men or not. As if that was like a goal I wanted to achieve with the therapy. And I think that was helpful. And it allowed me time to, like, reflect, and maybe have more like… Yeah, just reflect through my experiences and thoughts. **(P8, White British, Trams female, Sexual minority)**

Participants also spoke about the importance of therapists addressing these topics sensitively and according to their preferences. For example, participants noted that breaking up the discussion into multiple conversations helped them feel less overwhelmed with addressing these issues.

I say as an as and when it needs to be brought up because my [therapist] sees fit. And as we talked and I’ve talked to her previously, when there’s this thing that I’m not yet comfortable talking about it, we still can. We go on to the next thing rather than it being kind of continuous thing that we try and do in one session. We do it over a number of sessions. So it was helpful breaking it up. Breaking it up felt comfortable for me rather than talking about it for a long amount of time at once. **(P7, Black/Black British- Ethiopian, Cisgender female)**

Even when adaptations were not required as part of therapy, acknowledging and asking questions around the study terms allowed participants to build trust and feel validated. By engaging with these topics, participants felt that their therapists were open to understanding their unique identities and experiences.

Yeah, she [therapist] was attentive. She, like, brought it up in a few sessions.

And it felt like validating that she remembered that part of my identity, and that we could discuss it on multiple occasions. **(P8, White British, Trans female, Sexual minority)**

### Barriers to Exploring Diversity in Therapy

A prominent theme that emerged was barriers to discussing study terms as part of therapy. Some participants reflected on not discussing topics if the individual is not willing to. One participant spoke about barriers to exploring topics due to personal irrelevance. This emphasises the importance of being attuned to the individual’s willingness and readiness to explore certain issues.

You shouldn’t speak about something for the sake of talking about it if the person doesn’t want to. **(P4, White British, Cisgender female)**

For me personally, no. I just don’t feel the need to talk about it but that’s because religion isn’t important to me. **(P5, White British, Cisgender female, No religious affiliation)**

Participants highlighted the varied approaches to exploring diversity depending on the importance of these factors to the individual. Participants reflected that for individuals who have experienced hardship in relation to certain diversity factors, it may be more relevant to engage with such topics as part of therapy.

Everyone is different so it would really depend on the person. Like… I would think someone that’s faced more hardship because of like a certain identity…like I am gay and had shit for it so it affected me in that I wanted to talk about it in therapy, but this wouldn’t be the same thing for someone who was maybe straight and didn’t get shit for their sexuality. **(P3, White British, Cisgender female, Sexual minority)**

Negative past experiences and associated feelings were another reason why participants were resistant to exploring topics around the study terms. One participant noted that feeling judged and stereotyped for the things they shared led to them not being comfortable engaging with these topics. This highlights the need for therapists to consider the impact of past events on the individual’s engagement with therapy.

I think I wasn’t really keen on talking about it at first and um…it wasn’t because of [therapist], it was more so because of other times where um…when um…when I did bring it up or had talked about it, it was shut down or I was told I was being dumb for saying this and that so I guess I was worried the same thing would happen. **(P4, White British, Cisgender female, Sexual minority)**

## Discussions/Implications of the Study

The present study sought to understand YP’s experiences of therapy in relation to diversity considerations. Themes that emerged revealed both opportunities and obstacles for discussing diversity in therapy, providing insights into how adaptations and considerations can be made when supporting YP navigating these concerns.

One main theme revealed the importance of diversity in shaping YP’s identity and the ways they relate to others. Even when not directly impacting their personal experiences, YP still acknowledged the importance of these factors influencing social interactions and self-expression, both within and outside psychological therapy. Some also revealed an association between self-expression of certain identity factors such as faith, with varying degrees of mental wellbeing. Thus, therapists must move beyond the one-size fits all approach to being responsive and attuned to how these diverse factors shape YP’s experiences. This can be through being reflexive in their own approach but also holding space for YP to express and explore identities within therapy.

Another important theme highlighted the therapeutic relationship as being critical in YP’s engagement with conversations about diversity. For diverse YP, early and thoughtful inquiry into identity, context and lived experience is critical in building trust. These questions should be central in understanding presenting difficulties, therapeutic needs and barriers to accessing care. Moreover, therapists disclosing having faced similar concerns with previous clients reassured participants’ sense of their capacity to understand and appropriately support them. Akin to research evidence, adolescents’ perception of therapists was able to predict therapeutic outcomes (Tsamadou et al., 2021), and distrust of therapists is commonly reported as a barrier to therapy engagement (Stafford & Draucker, 2020; Uebelacker et al., 2012). Therapists need to prioritise creating a safe, non-judgemental space where YP can explore their identities and experiences. Ongoing professional development in competencies related to these issues allow for YP to feel confident in discussing these important aspects of their identity.

Findings from the study also revealed the importance of integrating diversity factors as part of therapy. YP expressed the importance of making adaptations to therapy where appropriate, based on the unique needs of the individual. For some, adaptations involved segmenting sensitive discussions around identity into more manageable parts to avoid them feeling overwhelmed. These findings emphasise the importance of flexibility and awareness in therapy (Owen & Hilsenroth, 2014). Being aware of these issues and bringing them to light can lead to fostering a sense of recognition and value, enhancing the therapeutic alliance.

The current study also identified a main theme of barriers to exploring diversity as part of the therapy. YP emphasised the significance of therapists understanding their readiness to engage with certain diversity topics. Discussions should be guided by the YP’s preferences and how relevant they feel certain diversity topics apply to them. Several participants with diverse identities noted feelings of judgement and stigma in previous mental health treatment. This limited their motivation to engage with discussions around these topics. Therapists need to consider the impact of past negative experiences, signal openness and invite conversations about diversity. These findings align with the broader implications of tailoring therapy to the individual’s preferences and experiences (Cooper et al., 2023; Gimeno-Peón, 2023; Zilcha-Mano et al., 2022).

Findings of this study provide insights into appropriate ways to discuss intersectionality and diversity during therapy. Therapist should be curious and open around diversity factors to gauge whether they are significant to the young person. This also offers opportunities for clinicians to be aware of systemic issues within healthcare and integrate social justice practices to the field. Research suggests that addressing and reducing systemic level inequality can contribute to greater overall wellbeing for marginalised individuals (Hatzenbeuhler et al., 2013; Kling et al., 2007). Considering intersecting identities as part of therapy therefore allows for potential biases within clinicians to be explored and addressed, thereby strengthening their professional practice. Thus, findings from the current study contributes to research evidence supporting clinicians to consider and address issues that affect the wellbeing of diverse individuals.

The present study was only successful at interviewing YP who had completed a course of therapy. This has implications for the findings as perspectives from YP who disengaged were not captured. Moreover, only one participant identified as male in the study (10%), thus further research is warranted to ensure that broader male perspectives are included within the evidence. Despite this, a large percentage of participants identified as being from an underrepresented sexual identity (70%). Thus, the current study contributes to the lack of research within underrepresented communities and provides valuable insights directly from diverse individuals.

As the current study is an extension of work previously completed by Sarr (2024), race/ethnicity was not included in the present study. Focusing on a few aspects related to diversity may therefore overlook the complex ways in which diverse identities shape a young person’s experiences. This is critical given research suggests that other diverse characteristics (e.g., education; socioeconomic status) can have an impact on mental health in children, particularly when there is an early onset (Reiss, 2013). Therefore, considering diversity as part of therapy requires the attention to all diversity factors relevant to the individual and their wellbeing. Further research is warranted to understand YP’s experiences of these diversity factors to better inform practice.

Although the terms were clearly defined, participants may have not fully understood the key terms or interpreted them in a different manner. This was recorded by three participants (30%) who had defined disability to include ASD. There is much debate about whether ASD is understood as a disability as not all individuals with ASD self-identify as being disabled (Lester et al., 2014). There is no clear consensus to consider ASD as a disability in research (Lester et al., 2014; Prins, 2019), therefore this was not included as part of the operationalised definition of the disability study term. Despite this, it is important to acknowledge that the issue of ASD as a disability emerged several times during interviews with participants. It cannot be predicted what might have been detected had ASD been explicitly addressed in this study, however it was meaningful for certain YP to acknowledge ASD as a disability and should be recognised as such. Further research including neurodiversity is warranted to better understand YP’s perspectives of diversity in therapy.

Differences between therapist and service users were briefly addressed in the current study. One participant spoke about being able to relate to their therapist who was from the same ethnic background. This mirrors findings from Sarr (2024) and suggest further research may benefit from exploring if perceived similarities/dissimilarities between YP and their clinician may impact the way these topics are brought up during therapy. Research also suggests that clinicians’ self-perception around diversity may impact the way these topics are addressed in therapy (Holyoak et al., 2020; Singer & Tummala-Narra, 2013). It may therefore be interesting to evaluate clinicians’ perspectives on discussing diversity during therapy and how they perceive their self-awareness, knowledge and skills around diversity.

The present study looked at YP’s perspective of discussing diversity factors (faith, sexual orientation, gender identity, disability) as part of psychological therapy. Results revealed several themes including (1) importance of these diversity factors in shaping YP’s identity and the ways they relate to others, (2) the therapeutic relationship, (3) integrating diversity in therapy and (4) barriers to exploring diversity in therapy. Findings of this study provide insights into appropriate ways to incorporate diversity during therapy. However, the limited focus on diversity characteristics warrants further research to better understand the complex and intersecting nature of these diversity characteristics. Nevertheless, findings of the current study demonstrate both opportunities and obstacles for acknowledging and addressing diversity in therapy, providing insights into how healthcare providers can tailor therapy when supporting YP.

## Supplemental Material


Supplemental Material - Young People’s Perspective on Discussing Intersectionality and Diversity During Psychological Therapy: A Qualitative Analysis in a Specialist Child and Adolescent Mental Health Service
Supplemental Material for Young People’s Perspective on Discussing Intersectionality and Diversity During Psychological Therapy: A Qualitative Analysis in a Specialist Child and Adolescent Mental Health Service by Hanan K. S. Khalaf, Nada Abou Seif, Zoë Maiden in Clinical Child Psychology and Psychiatry
