# Supplementary material for: Young People’s Perspective on Discussing Intersectionality and Diversity During Psychological Therapy: A Qualitative Analysis in a Specialist Child and Adolescent Mental Health Service
Source: Clin Child Psychol Psychiatry. 2026 Jan 20;31(2):337–51. doi: 10.1177/13591045261419862 (PMC12992633; doi:10.1177/13591045261419862)
Supplement: Supplemental Material - Young People’s Perspective on Discussing Intersectionality and Diversity During Psychological Therapy: A Qualitative Analysis in a Specialist Child and Adolescent Mental Health Service [file sj-pdf-1-ccp-10.1177_13591045261419862.pdf]

Supplementary materials: Interview guide

Adolescent Interview Guide:

Introductions (of interviewer and of terms used)

My name is X and I am a trainee psychologist at the TAD clinic. We would like to find out about your experience at the TAD clinic, particularly in relation to diversity. You can choose not to answer any of the questions asked throughout this interview and you don't have to give a reason for not answering. Before we start with the interview questions, I just want to check:

1. How old are you?
2. How would you describe your religion/faith?
    - *For example: I'm X or I practice X.*
    - *Note: this can include atheism.*

I'm now going to ask you about some terms. You may already know what these mean, but I'm going to ask just to ensure that we are on the same page.

3. Have you heard the term gender identity before?
    - If "yes":
        - What does it mean to you?
        - It's a way to describe how someone feels about their gender. For example, some people may identify as a boy or a girl, while others may find neither of these terms feel right for them and identify as neither or somewhere in the middle. This may be different from someone's biological sex.
    - If "no":
        - It's a way to describe how someone feels about their gender. For example, some people may identify as a boy or a girl, while others may find neither of these terms feel right for them and identify as neither or somewhere in the middle. This is different from someone's biological sex.
        - Do you understand? Do you have any questions?
    - How would you describe your gender identity?

4. Have you heard the term sexual orientation before?
    - If "yes":
        - What does it mean to you?
        - It's a way to describe who someone feels physically and emotionally attracted to. This can be romantic or emotional attraction, or both.
    - If "no":
        - It's a way to describe who someone feels physically and emotionally attracted to. This can be romantic or emotional attraction, or both.
        - Do you understand? Do you have any questions?
    - How would you describe your sexual orientation?

5. Have you heard the term disability before?
    - If "yes":
        - What does it mean to you?
        - It's when someone has significant difficulties with communication, comprehension, vision, hearing or physical functioning.
    - If "no":
        - It's when someone has significant difficulties with communication, comprehension, vision, hearing or physical functioning.

    - Do you understand? Do you have any questions?
- Do you have a disability? This can be either diagnosed or undiagnosed.
    - If yes, could you describe it.
    - [<u>If no, do not ask about disability within the interview</u>]

Now, I will ask you some questions about your experience at the clinic, and it should take around 30 minutes. We can take a break at any time. [Confidentiality explanation – e.g., your therapist won't know about the answers, and the results will be anonymised when a report is written]. Before we start, do you have any questions for me?

*For each definition, have it in front of the young person (e.g., screen share or on a piece of paper).

[Start recording]

<u>**Interview Questions [for each of sections A – D, only continue with Q2 – Q7 if the YP has identified the aspect of diversity as being important to them in Q1. If they answer "no" to Q1 of each section (i.e. A1, B1, C1, D1), move to the next section].**</u>

A. Religion/Faith

1. Is your religion/faith important to you? [yes/no]
    - *Note: this can include atheism.*
    - If "yes", why is it important to you?
    - *[Interviewer note: If 'no', move to section B]*
2. Was your religion/faith asked about in this clinic?
    - If "yes", when? (e.g. registration form, assessment, therapy)?
3. Did your therapist pay attention to your religion/faith during therapy at the TAD clinic by:
    - Having curiosity about your religion (e.g., were you asked about what your religion/faith meant to you?)
    - Making changes for you (e.g., changing session time due to Ramadan or changing session time due to Yom Kippur)?
4. When making sense of your difficulties, was religion/faith talked about?
5. When talking about strategies to get better, was your religion/faith talked about (e.g., religion as a source of support)?
6. Are you happy with how religion/faith was talked about? [Yes/No]
7. If you were to be asked:
    - When would you prefer to talk about your religion/faith (e.g., questionnaire, in the assessment interview, therapy, talking about it openly, not talk about it)?
    - How would you like it to be brought up (e.g., asked about it directly, not raised unless brought up by you)?
    - What would you want your therapist to do or keep in mind when talking about religion/faith (e.g., trying to be understanding, being curious, validate experiences)?
    - Talking about current matters which could relate to you?

B. Gender Identity

1. Is your gender identity important to you? [yes/no]
    - If "yes", why is it important to you?
    - *[Interviewer note: If 'no', move to section C]*
2. Was your gender identity asked about in this clinic?
    - If "yes", when? (e.g. registration form, assessment, therapy)?
3. Did your therapist pay attention to your gender identity by:
    - Having curiosity about your gender identity (e.g., were you asked about your pronouns?)
    - Making changes for you (e.g., using your preferred pronouns)?
4. When making sense of your difficulties, was gender identity talked about?
5. When talking about strategies to get better, was your gender identity talked about?
6. Are you happy with how gender identity was talked about? [Yes/No]

7. If you were to be asked:
    - When would you prefer to talk about your gender identity (e.g., questionnaire, in the assessment interview, therapy, talking about it openly, not talk about it)?
    - How would you like it to be brought up (e.g., asked about it directly, not raised unless brought up by you)?
    - What would you want your therapist to do or keep in mind when talking about gender identity (e.g., trying to be understanding, being curious, validate experiences)?
    - Talking about current matters which could relate to you?

C. Sexual Orientation

1. Is your sexual orientation important to you? [yes/no]
    - If "yes", why is it important to you?
    - *[Interviewer note: If 'no', move to section D]*
2. Was your sexual orientation asked about in this clinic?
    - If "yes", when? (e.g., registration form, assessment, therapy)?
3. Did your therapist pay attention to your sexual orientation by:
    - Having curiosity about your sexual orientation?
    - Making changes for you?
4. When making sense of your difficulties, was sexual orientation talked about?
5. When talking about strategies to get better, was your sexual orientation talked about?
6. Are you happy with how sexual orientation was talked about? [Yes/No]
7. If you were to be asked:
    - When would you prefer to talk about your sexual orientation (e.g., questionnaire, in the assessment interview, therapy, talking about it openly, not talk about it)?
    - How would you like it to be brought up (e.g., asked about it directly, not raised unless brought up by you)?
    - What would you want your therapist to do or keep in mind when talking about sexual orientation (e.g., trying to be understanding, being curious, validate experiences)?
    - Talking about current matters which could relate to you?

D. Disability [only ask if YP identifies as having a disability]

1. Is your disability status important to you? [yes/no]
    - If "yes", why is it important to you?
    - *[Interviewer note: If 'no', move to section E]*
2. Was your disability asked about in this clinic?
    - If "yes", when? (e.g. registration form, assessment, therapy)?
3. Did your therapist pay attention to your disability by:
    - Having curiosity about your disability (e.g., were you asked about your disability?)
    - Making changes for you?
4. When making sense of your difficulties, was disability talked about?
5. When talking about strategies to get better, was your disability talked about?
6. Are you happy with how disability talked about? [Yes/No]
7. If you were to be asked:
    - When would you prefer to talk about your disability (e.g., questionnaire, in the assessment interview, therapy, talking about it openly, not talk about it)?
    - How would you like it to be brought up (e.g., asked about it directly, not raised unless brought up by you)?
    - What would you want your therapist to do or keep in mind when talking about disability (e.g., trying to be understanding, being curious, validate experiences)?
    - Talking about current matters which could relate to you?

E. General

1. What was the best part about your experience with TAD?

- If the YP indicates a specific person (e.g., their therapist), what did they do?

2. What didn’t you like about it?
3. Is there anything you think we should do differently when working with young people with diverse characteristics?
4. How has this interview felt for you? (e.g., comfortable/uncomfortable to speak about?)

Those are all the questions I have. Do you have any questions for me or is there anything I didn’t ask you about that you would like to add? Thank you for your time today
